# Antimicrobial and Antiviral Activities, and Biocompatibility of Titanium Coated with Electron Beam-Irradiated α‑Silver Tungstate Microcrystals

**DOI:** 10.1021/acsomega.5c07850

**Published:** 2025-11-23

**Authors:** Sarah Raquel de Annunzio, Marcelo Assis, Paula Aboud Barbugli, Alice Santos Rosa, Thamara Kelcya Fonseca Oliveira, Tayane Alvites Nunes Quintão, Vivian Neuza dos Santos Ferreira, Thayane da Encarnação Sá-Guimarães, Giovanna Barbosa da Conceição, Débora Ferreira Barreto-Vieira, Rodolfo Debone Piazza, Rodrigo Fernando Costa Marques, Milene Dias Miranda, Elson Longo, Carlos Eduardo Vergani

**Affiliations:** A Departamento de Materiais Odontológicos e Prótese, Faculdade de Odontologia, 153998 Universidade Estadual Paulista “Júlio de Mesquita Filho” (UNESP), Araraquara, SP 14801-903, Brazil; B Departamento de Biociências, Universidade Federal de São Paulo (UNIFESP), Santos, SP 11015-020, Brazil; C 37903 Laboratório de Morfologia e Morfogênese Viral, Instituto Oswaldo Cruz, Fundação Oswaldo Cruz, Rio de Janeiro, RJ 21040-900, Brazil; D Departamento de Química Analítica, Físico-Química e Inorgânica, Instituto de Química, 28108 Universidade Estadual Paulista “Júlio de Mesquita Filho” (UNESP), Araraquara, SP 14800-060, Brazil; E 67828 CDMF, Universidade Federal de São Carlos (UFSCar), São Carlos, SP 13565-905, Brazil

## Abstract

The development of biomaterials with modified surfaces is a promising strategy to prevent microbial adhesion, biofilm formation, and viral contamination on implants. Alpha-silver tungstate (α-Ag_2_WO_4_) exhibits well-recognized antimicrobial activity, which can be further enhanced by electron beam irradiation (EBI). This study evaluated the antimicrobial, antiviral, and cytotoxic performance of titanium (Ti) discs coated with α-Ag_2_WO_4_, with or without EBI treatment, using the spin coating method. Material characterization revealed no significant differences in structure, composition, or morphology between the two coatings. Both coatings increased the surface roughness and surface free energy of the discs. Regarding antibiofilm effects, reductions of 3.46 and 3.31 log_1__0_ CFU/mL were observed for *Streptococcus sanguinis* on irradiated and nonirradiated coated surfaces, respectively, whereas *Candida albicans*, *Actinomyces naeslundii*, *Fusobacterium nucleatum*, and *Porphyromonas gingivalis* showed no growth after the adhesion and biofilm formation phases. Furthermore, the α-Ag_2_WO_4_ coatings reduced SARS-CoV-2 titers by up to 2 log_1__0_ without causing cytotoxic effects in Vero E6 cells. These findings underscore the potential of α-Ag_2_WO_4_-based coatings as effective antimicrobial and antiviral surfaces, offering promising applications in dental implants and other biomedical devices.

## Graphical abstract

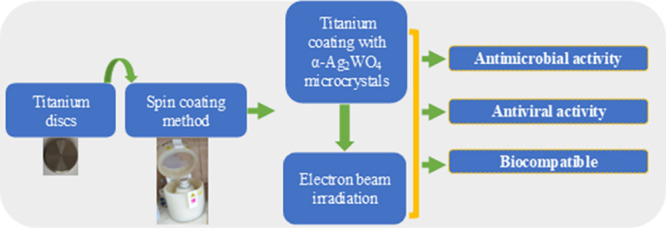

## 1 Introduction

Peri-implantitis is a polymicrobial infection that occurs within implant sites due to contamination by microorganisms and poor oral hygiene. Here, biofilms form, causing inflammatory lesions in tissues and alveolar bone losses.^1–3^ In a previous work conducted by Elani et al. (2018),^4^ the authors projected that in the United States, the prevalence of dental implants could reach up to 23% by 2026. Another work conducted by Ronandini et al. (2021)^5^ found that 56.6% of patients experienced peri-implantitis after at least one year after dental implant placement.

Biofilm formation on dental implant structures begins with the adhesion of a protein film to the dental implant surface, which is followed by initial adhesion and colonization of Gram-positive streptococci, including *Streptococcus sanguinis* and anaerobic cocci, and *Actinomyces* species, including *Actinomyces naeslundii*, as primary colonizers. The formation and maturation of polymicrobial biofilms on implant structures are enabled by *Fusobacterium nucleatum*, since it coaggregates with numerous oral bacterial species and serves as a bridge between the primary and secondary colonizers. Secondary colonizers are Gram-negative facultative anaerobic rods such as *Aggregatibacter actinomycetemcomitans* and strict anaerobes such as *Porphyromonas gingivalis*, *Treponema denticola*, *Tannerella forsythia*, and *Prevotella intermedia*.^6–8^

Among secondary colonizers, *P. gingivalis* is the most prevalent microorganism found in peri-implantitis; its pathogenic role in disease progression depends mainly on its ability to adhere to host cells and coaggregate with other bacterial species in the subgingival biofilm.^9,10^ In addition to these periodontal pathogens, *Candida albicans* is considered an opportunistic species in peri-implant lesions.^11,12^ Implant surfaces provide the necessary substrates for the formation of fungal biofilms and thus can be considered a potential reservoir for (re)­infection with oral *C. albicans*, which may contribute to the prevalence of oral candidosis in predisposed patients. Additionally, SARS-CoV-2 has been found in saliva and has already been identified in the gingiva and gingival crevicular fluid of patients with COVID-19.^13,14^ Thus, approaches to control the growth of these pathogens and inhibit the adhesion and formation of bacterial and fungal biofilms on the surface of implants are of great clinical relevance.^15,16^

In recent years, researchers have dedicated themselves to the development of dental materials coated with antimicrobial agents,^17–23^ including by coating dental-medical devices, including abutments.^24,25^ Recently, several approaches have been described, such as the incorporation of nanoparticles and bioactive polymers in combination with photothermal therapy, which can promote antibacterial effects associated with immune modulation and osteogenesis.^26^ Additionally, mesoporous silica nanoparticles loaded with antimicrobial peptides have been reported to enable sustained release of the peptides, biofilm inhibition, and induction of osteogenic differentiation.^27^ Other studies have explored biomimetic surfaces enriched with metal ions, such as copper and silver (Ag), which combine antimicrobial activity with biocompatibility.^28,29^ Another strategy involves the use of nanoparticle coatings with redox activity capable of inducing ferroptosis,^30^ as well as Ag deposition methods via ion plating.^31^ More recently, pH-responsive coatings with biocompatible properties and corrosion resistance, designed to respond to infectious microenvironments, have gained attention for their selective release of antimicrobial peptides.^32^ Furthermore, nanomaterials from the MXene class have demonstrated biocompatible, antimicrobial, and antiviral properties, including activity against SARS-CoV-2.^33^ Despite these advances, there is still a pressing need for strategies that effectively combine antimicrobial and antiviral activity while maintaining biocompatibility.

Pure Ti and its alloys are commonly used for implant abutments because they present good biocompatibility and osseointegration.^34^ Among the coating methods used, spin coating presents several benefits, as it is a simple, efficient, reproducible, scalable, low-cost methodology that can be applied to solid substrates, such as Ti, and allows precise control over the thickness of the deposited film.^35^ Unlike more complex vacuum-based processes, spin coating requires relatively simple equipment and mild processing conditions, which facilitates rapid prototyping and experimental exploration of multifunctional Ti coatings. Furthermore, the ability to generate homogeneous, defect-free surfaces makes this approach particularly suitable for systematic studies on the antimicrobial and antiviral properties of modified Ti substrates.^35^ Previous studies have demonstrated that spin coating Ti can generate bioactive surfaces with antimicrobial effects without compromising biocompatibility.^36,37^ Despite these advances, only a limited number of studies have simultaneously assessed the antimicrobial and antiviral potential of such Ti coatings.

In recent years, our research group has conducted several studies on the antimicrobial properties of a variety of inorganic materials.^38–42^ Among them, silver tungstate (Ag_2_WO_4_) has shown promising properties. Studies conducted by our group reported effective antifungal and bactericidal activity of α-Ag_2_WO_4_ against the most prevalent microorganisms in the oral cavity, as well as anti-SARS-CoV-2 activity.^40,43–48^ Ag_2_WO_4_ is appropriate to use as it is nontoxic to several types of cells and tissues *in vitro*.^49,50^ This microcrystal inactivates microorganisms by the formation of reactive oxygen species (ROS) and the ionic release of silver (Ag^+^) and tungsten (W^6+^) ions.^47,51^ This activity can be further enhanced by EBI of the Ag_2_WO_4_ powder, mainly due to the formation of metallic silver nanoparticles (Ag NPs) on its surface and other three-dimensional structural modifications.^52,53^ This improvement in the antimicrobial activity of Ag_2_WO_4_ in its alpha (α) phase, achieved through EBI, was reported against fungi and bacteria of significant medical-dental relevance.^51–53^ In this context, the present study aimed, for the first time, to deposit α-Ag_2_WO_4_ onto Ti discs using the spin coating method, irradiate the samples with an EBI system, and evaluate their antimicrobial and antiviral activities, as well as their biocompatibility.

## 2 Experimental Section

### 2.1 Synthesis of α-Ag_2_WO_4_ Microcrystals

α-Ag_2_WO_4_ microcrystals were synthesized by the coprecipitation method, as previously described by Foggi et al. (2017).^44^ To obtain the microcrystals, 2 × 10^–3^ mol silver nitrate (AgNO_3_; 99.98% purity (Cennabras, Guarulhos, SP, Brazil) was diluted in 50 mL of deionized water. At the same time, 1 × 10^–3^ mol of sodium tungstate dihydrate was dissolved separately in 50 mL of water (Na_2_WO_4_.2H_2_O; 99.99% purity; Sigma-Aldrich, St. Louis, MO, USA). Both solutions were kept heated and magnetically stirred. When the solutions reached 70 °C and were fully mixed, a precipitate formed. This precipitate was then washed 5 times with distilled water for 1 min at 600 rpm to remove residual ions and then dried at 60 °C.

### 2.2 Coating of Ti Discs with α-Ag_2_WO_4_

Grade 2 Ti alloy discs (Realum, São Paulo, SP, Brazil) measuring 11 mm in diameter and 3 mm in thickness were used here, exhibiting an average standard surface roughness of 0.2 μm ± 0.01.^17^ To deposit the α-Ag_2_WO_4_ onto the Ti disc surface, a solution of polyethylene glycol (PEG 300, molecular weight 300 g/mol, Sigma -Aldrich), with 10 mg of α-Ag_2_WO_4_ for each 1 mL of PEG 300 used, was continuously stirred at 700 rpm at 25 °C to avoid settling of the microcrystals. 30 μL of this solution was carefully spotted onto the center of each disc, and the disc was placed on the spin coater (model WS 650 HZ - 23 NPPB/OND 0, Laurell Technologies Corporation - North Wales, PA, USA). Then, the disc was rotated at 2000 rpm in a vacuum for 30 s, removed, and dried at 90 °C. This procedure was performed 3 times on both sides of the discs. After drying, the samples were kept at 300 °C for 30 min. To analyze the amount deposited on each disc, weighing was performed before and after the thermal fixation process and the average amount of material deposited on the entire surface of the disc was 0.01 g.

### 2.3 Electron Beam Irradiation (EBI)

After coating, the discs were irradiated by an EBI system, as described by da Costa et al. (2021),^54^ with portable electron irradiation equipment, with an accelerating voltage of 40 kV, 10^–3^ mTOrr and 15 mA, for 5 min on each surface. The working area was 5 cm.

### 2.4 Characterizations

The information on the crystal structures and the structural organization of the materials was determined by XRD measurements as described by Assis et al. (2021).^41^ For this, a D/Max-2500PC diffractometer (Rigaku, Japan) utilizing Cu Kα radiation (λ = 1.5406 Å) between 10 and 110° (2θ) was used. The crystal structure and the short-range organization of the deposited materials were investigated by micro-Raman spectroscopy on a LabRAM iHR550 spectrometer (Horiba Jobin-Yvon, Japan). This was equipped with a charge-coupled device (CCD), using a He–Ne laser (MellesGriot, United States) with a wavelength of 632.8 nm.^41^ The surface morphology of the deposited films was determined by SEM on a TM4000 II benchtop microscope (Hitachi, Japan), equipped with an EDS detector that allows mapping the chemical composition of the samples. The experimental groups evaluated were 1) **Ti:** uncoated titanium, 2) **Ti–I:** uncoated titanium with EBI, 3) **Ti α-Ag**_**2**_ **WO**_**4:**_ titanium coated with α-Ag_2_WO_4_, and 4) **Ti α-Ag**_**2**_ **WO**_**4**_ **-I:** titanium coated with α-Ag_2_WO_4_ and underwent EBI.

### 2.5 Wettability and Surface Free Energy

The wettability and surface free energy of the deposited materials were investigated through contact angle measurements. The tests were conducted on an OCA-20 Goniometer from DataPhysics Instruments GmbH. Static contact angle measurements were collected using the sessile drop method at 25 °C using distilled water (10 μL), diiodomethane (2 μL), and formamide (7 μL). Here, each Ti disc was carefully positioned on a table between the camera and the lens before dispensing a drop onto the disc. After allowing 3 s for the drop to stabilize, the contact angles formed between the surface of each sample and the liquid drop were calculated using the Laplace–Young equation in triplicate (n = 9 for all three liquids). The surface free energy was then calculated using the concept of polar components and dispersion with the Owens–Wendt–Rabel–Kaelble (OWRK) method.^55^

### 2.6 Surface Roughness

The average surface roughness (Ra/μm) of the deposited films was determined by a profilometer (Mitutoyo SJ 400; Mitutoyo Corporation, Tokyo, Japan) with an accuracy of 0.01 μm, an interval (cutting length) of 0.8 mm, a transverse length of 2.5 mm, a stylus speed of 0.5 mm/s, and a tip radius of 5 μm, as described by de Foggi et al. (2016).^56^ Four readings were taken for each sample, at different locations, within a standardized area for all samples in triplicate at three independent times (n = 9).

### 2.7 Antibacterial and Antifungal Activity

#### 2.7.1 Human Saliva Preparation and Formation of the Salivary Pellicle on the Discs

This study was approved by the Research Ethics Committee of São Paulo State University (Unesp) School of Dentistry (CAAE: 68290823.7.0000.5416). The protocol used was that as described by Garcia de Carvalho et al. (2020).^57^ Collected saliva was centrifuged at 10,000 rpm for 15 min at 4 °C (Eppendorf, Hamburg, Germany), filtered through a 0.22 μm membrane (Kasvi, PR, Brazil), and then stored at −80 °C until use. To mimic the microenvironment of the oral cavity, each disc was placed into 300 μL of the filtered human saliva in a 24-well plate using sterile forceps and incubated in an orbital shaker at 37 °C (75 rpm) for 30 min. Afterward, the saliva was aspirated from each well and discarded before dispensing 1 mL of the standardized microorganism inoculum.

#### 2.7.2 Strains and Culture Conditions

The assays were performed using standard strain of *Candida albicans* (American Type Culture Collection - ATCC 90028), *Streptococcus sanguinis* (ATCC 10556), *Actinomyces naeslundii* (ATCC 12104), *Fusobacterium nucleatum* (ATCC 25586) and *Porphyromonas gingivalis* (ATCC 33277). When required, *C. albicans* was streaked onto Sabouraud Dextrose Agar (SDA, Himedia, Mumbai, India) at 37 °C for 48 h; *F. nucleatum* and *P. gingivalis* onto anaerobic agar at 37 °C under anaerobiosis for 3 and 5 days, respectively; *S. sanguinis* on Brain Heart Infusion Agar (Kasvi, PR, Brazil) at 37 °C with 5% CO_2_ for 48 h; and *A. naeslundii* on blood agar plates with 5% sheep blood at 37 °C with 5% CO_2_ for 48 h. The preinoculum and inoculum of *C. albicans* were cultured in Yeast Nitrogen Base broth (YNB - Himedia, Mumbai, India) with dextrose, *F. nucleatum* and *P. gingivalis* in Fastidious Anaerobe Broth (FAB - Neogen, Michigan, USA), and *S. sanguinis* and *A. naeslundii* in Tryptic Soy Broth (TSB - Kasvi, PR, Brazil). Each strain was cultured according to a previously established growth curve until they reached the mid log growth phase before adjusting the cell concentration in a spectrophotometer to a concentration of 1 × 10^6^ CFU/mL for *C. albicans* and 1 × 10^7^ CFU/mL for the bacteria.

#### 2.7.3 Evaluation of the Antimicrobial Activity of Ti Discs at the Adhesion and Biofilm Phases by Colony-Forming Units per Milliliter (CFU/mL)

First, a sterile swab was used to remove excess α-Ag_2_WO_4_ that had not adhered to the surface of the Ti discs before washing them with PBS and transferring them to 24-well plates. Next, the saliva pellicle was formed as described in Section 2.7.1, and the biofilms were formed as described below.

For the *C. albicans* biofilms formation, 1 mL of the fungal suspension in Roswell Park Memorial Institute broth (RPMI-1640; Sigma-Aldrich) was added to the discs and the samples were incubated at 37 °C under shaking at 75 rpm for 1.5 h (adhesion period).^58^ After this period, the discs were carefully washed twice with PBS and 1 mL of RPMI-1640 was added to each well. After 24 h, the culture medium was renewed and the samples were kept in incubation for another 24 h (totalling 48 h of biofilm formation). *S. sanguinis* and *A. naeslundii* were formed at 37 °C with 5% CO_2_. The adhesion phase was also 1.5 h.^59^ After this period, the culture medium (TSB) was removed and the discs were washed twice with PBS. After, 1 mL of TSB was added to each sample. After 24 h, the culture medium was renewed and the samples were kept in incubation for another 24 h. For the *F. nucleatum* and *P. gingivalis* biofilm formation, the adhesion phase was 24 h, and the biofilm was formed during 7 days. The FAB culture medium was changed daily, until the seventh day.^16,60^ For the CFU/mL quantification the discs were transferred to wells containing 1 mL of PBS and carefully scraped. This content was transferred to sterile microtubes, homogenized by vortex, and finally the samples were diluted to 10^–4^ for *C. albicans* and 10^–5^ for the bacteria. Next, 10 μL were plated on agar in duplicate using the drop method. The colonies grown were counted after 24 h of growth for *C. albicans*, *S. sanguinis* and *A. naeslundii* and 7 days for *F. nucleatum* and *P. gingivalis*. The tests were performed in triplicate on three different occasions (n = 9).

#### 2.7.4 LIVE/DEAD Assay by Confocal Laser Scanning Microscopy (CLSM)

The viability of the microorganisms adhered to Ti discs was performed using the LIVE/DEAD BacLight Bacterial Viability Staining Kit (Invitrogen, CA, USA) according to the manufacturer’s instructions. Briefly, Syto-9 and Propidium Iodide (PI) (1:1000) were added to each sample for 30 min, then washed with PBS. The images were acquired using a Carl Zeiss LSM 800 microscope with Airyscan (Carl Zeiss, Germany) at a 10× magnification objective. The laser ranges used for detection were 488 to 520 nm for Syto-9 and 561 to 620 nm for PI.^60^ Three equidistant fields of each sample were evaluated.

### 2.8 Virucidal Activity

The virucidal efficacy of the metal discs **Ti**, **Ti–I**, **Ti α-Ag**_**2**_ **WO**_**4**_, and **Ti α-Ag**_**2**_ **WO**_**4**_ **-I** (measuring 5 mm in diameter and 2 mm in thickness) was assessed against SARS-CoV-2 B1 lineage, GenBank accession MT710714, SisGen-Brazil AC58AE2 at a concentration of 10^4^ plaque-forming units per milliliter (PFU/mL). The viral samples were incubated either in Dulbecco’s Modified Eagle Medium (DMEM, Gibco), as a control, or with the specified metal discs for 5, 20, 40, or 60 min at room temperature. Postincubation, the supernatants were serially diluted (ranging from 1:10 to 1:1280) and subsequently applied to a 96-well plate seeded with Vero E6 cells (ATCC CRL-1586), an epithelial cell line derived from the kidney of the African green monkey, at a density of 10^4^ cells per well.

Following a 1-h incubation at 37 °C containing 5% CO_2_ to facilitate viral entry and the initial infection, the cells were overlaid and incubated in a medium composed of DMEM-High Glucose 10x supplemented with 2.4% carboxymethylcellulose, 2% Fetal Bovine Serum (FBS, Gibco), and 1% penicillin/streptomycin for 72 h. Afterward, the cell cultures were fixed using 4% formalin for 3 h and subsequently stained with 0.04% crystal violet for 1 h to facilitate the quantification of plaque-forming units (PFU), thereby determining the viral titers.

It is imperative to note that all procedures involving the virus were conducted in a biosafety level 3 facility, adhering strictly to the guidelines recommended by the World Health Organization (WHO).

### 2.9 Vero E6 Viability

To ascertain the cytocompatibility of the supernatants derived from exposure to various metal discs, cytotoxicity assays were conducted on Vero E6 cells using discs **Ti**, **Ti–I**, **Ti α-Ag**_**2**_ **WO**_**4**_, and **Ti α-Ag**_**2**_ **WO**_**4**_ **-I**. These discs were incubated in DMEM supplemented with 10% FBS and 1% penicillin/streptomycin for 1 h at ambient temperature. After incubation, the resulting supernatants were transferred to 96-well plates containing Vero E6 cells at a density of 1 × 10^4^ cells per well and were cultured for 72 h at 37 °C in an atmosphere containing 5% CO_2_. Cell viability was assessed using the MTT (3-(4,5-Dimethyl-2-thiazolyl)-2,5-diphenyl-2H-tetrazolium Bromide, Sigma-Aldrich) assay, following the manufacturer’s instructions. Briefly, 10 μL/well of MTT 5.0 mg/L solution was added to the plaque and incubated for 2 h before adding 10% SDS and incubating for another 2 h. The spectrophotometric absorbance analysis was carried out at 570 nm.^61^ The result is expressed as the percentage of viable cells, based on the calculation:

$$\mathrm{cell}\;\mathrm{viability}=100-\frac{\left[(\mathrm{MOCK}-\mathrm{treatment})\times 100\right]}{\mathrm{lysis}\;\mathrm{control}}$$

### 2.10 Negative Staining Technique of SARS-CoV-2 Particles for Analysis by Transmission Electron Microscopy (TEM)

Metallic discs (**Ti**, **Ti–I**, **Ti α-Ag**_**2**_ **WO** **4**, **Ti α-Ag**_**2**_ **WO**_**4**_ **-I**) were exposed to SARS-CoV-2 viral cultures at a concentration of 10^6^ PFU/mL and incubated under ambient conditions for 1 h. Throughout this incubation period, the mixtures were agitated manually at 10 min intervals to ensure homogeneity. Following the incubation, the supernatant was harvested and aliquoted; 10% was preserved via freezing for subsequent viral titer quantification using PFU assays, while the remaining 90% was prepared for TEM by dilution in 2.5% glutaraldehyde within a 0.1 M sodium cacodylate buffer at pH 7.2.

For the TEM analysis, a single drop of the virus suspension was placed on a Formvar-coated 400-mesh copper grid (Electron Microscopy Sciences, Hatfield, PA, USA). After 25 s the surplus material was removed using filter paper, and subsequently, a drop of 2% phosphotungstic acid (PTA), buffered at pH 7.0, was applied. The excess PTA was blotted away after 15 s before treating the samples with a 1% glutaraldehyde solution in sodium cacodylate buffer (1:1 v/v) to comply with biosafety level requirements. The grid was then examined under a Hitachi HT 7800 transmission electron microscope (Hitachi, Chiyoda, Tokyo, Japan).^62–64^ To comply with biosafety level requirements, the SARS-CoV-2 samples were treated with a 1% glutaraldehyde solution in sodium cacodylate buffer (1:1 v/v) to inactivate the virus prior to grid preparation and TEM observation.

### 2.11 Statistical Analysis

Data from surface roughness, antibacterial, and antifungal activity measurements were analyzed using the Kruskal–Wallis test, followed by Dunn’s post hoc test. For contact angle measurements, a one-way ANOVA followed by Tukey’s post hoc test was applied. For antiviral activity assays, a one-way ANOVA followed by Tukey’s post hoc test was applied for single time-point data, whereas a two-way ANOVA followed by Tukey’s post hoc test was used for data involving two or more time points. All analyses were performed using GraphPad Prism software (GraphPad Software Inc., La Jolla, CA, USA). A significance level of 5% (*p* < 0.05) was adopted for all statistical tests.

## 3 Results and Discussion

### 3.1 Characterizations

The XRD analysis showed that the α-Ag_2_WO_4_ microcrystals obtained by the coprecipitation method had an orthorhombic structure, with a space group of *Pn2n* (Figure 1), with diffraction peaks in agreement with JCPDS document No. 34–61. In addition, no secondary phases were observed in the material. The Ti discs, on the other hand, had a hexagonal structure, with a space group of *P63/mmc*, with diffraction peaks in agreement with JCPDS document No. 44–1294. Both of these phases can be observed when depositing the α-Ag_2_WO_4_ on the surface of the Ti (Ti α-Ag_2_WO_4_) disc (Figure 1A). Furthermore, as the Ti disc undergoes heat treatment, it is also possible to verify the formation of titanium dioxide (TiO_2_) (rutile) with a tetragonal structure, with a space group of *P42/mnm*. No significant differences were observed between the diffractograms after irradiation (Ti α-Ag_2_WO_4_–I) (Figure 1B).

**Figure 1:**
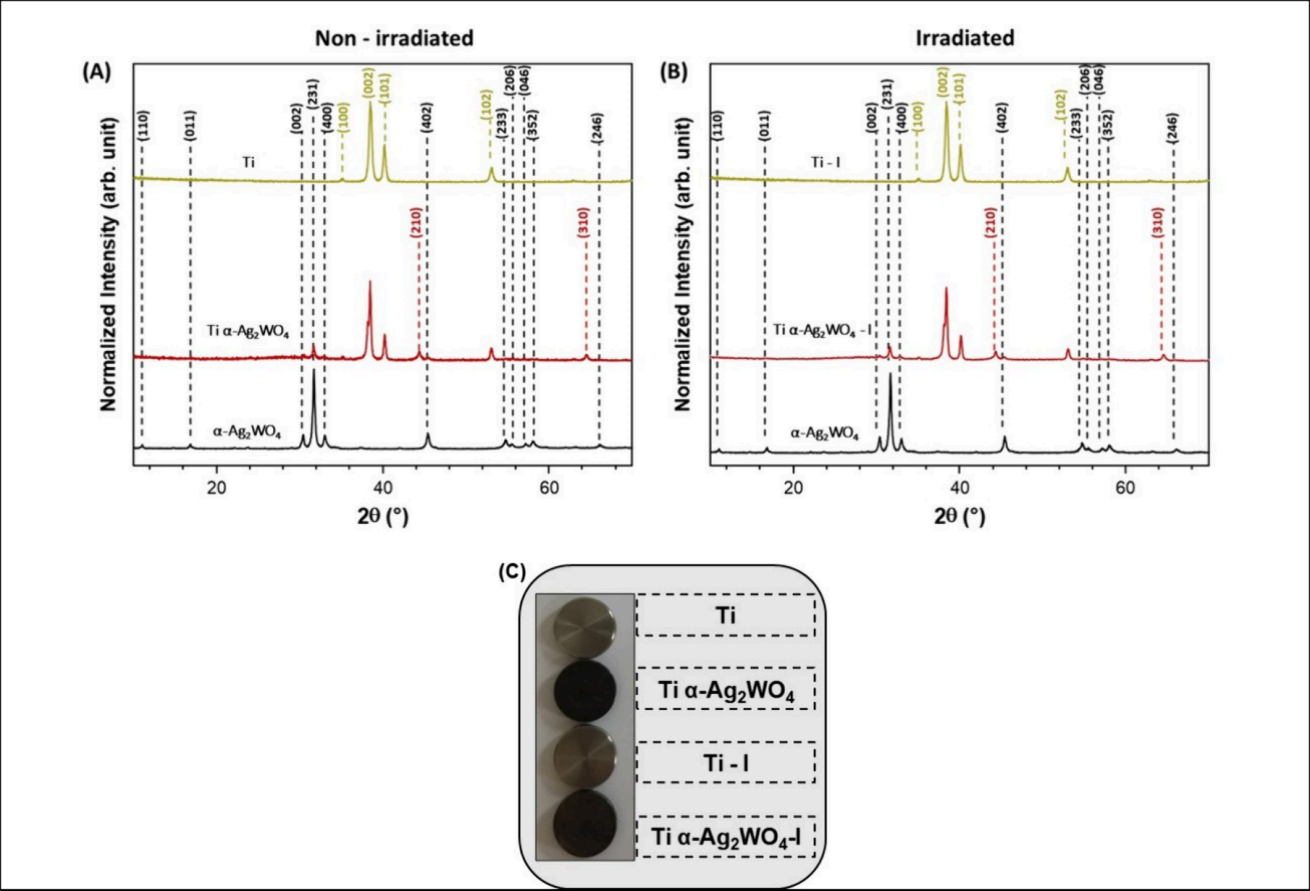
XRD of nonirradiated (A) and irradiated Ti discs (B) and Ti discs (C).

Characteristic Raman vibrational modes were also observed for the Ti α-Ag_2_WO_4_ and Ti α-Ag_2_WO_4_–I discs (Figure S1). For Ti, it was not possible to observe Raman scattering peaks, but the samples with α-Ag_2_WO_4_ had the characteristic peaks of the orthorhombic structure of α-Ag_2_WO_4_ at 138.6, 277.7, 342.1, 700.3, 798.5, and 887.6 cm^–1^. The modes located below 300 cm^–1^ are associated with A_2g_ (138.6 cm^–1^) and B_1g_ (277.7 cm^–1^) transitions of the crystal lattice. Vibrations of the cationic lattice and stretching of the W–O bonds are noted at the modes located at 342.1 (B_2g_) and 700.3 cm^–1^ (B_1g_), respectively. In addition, the modes located at 798.5 (A_2g_) and 887.6 cm^–1^ (A_1g_) correspond to symmetric and asymmetric stretching of the O–W–O bonds in the [WO_6_] clusters, respectively.^41^ The profiles were similar in both cases, with no differences in the Ti α-Ag_2_WO_4_ and Ti α-Ag_2_WO_4_–I discs. This behavior was previously demonstrated by Assis et al. (2019)^51^ and Assis et al. (2018)^65^ and used as the XRD and Raman spectroscopy techniques do not have sufficient sensitivity to show significant structural changes after the EBI process.

Since the structural characteristics of α-Ag_2_WO_4_ were maintained in both coatings, further analyses were conducted to evaluate their impact on the surface of the Ti discs. In Figure 2A–D, it is evident that the pure Ti disc only shows texturing from machining and subsequent sanding treatment. In contrast, for the coated Ti discs, uniformly distributed microrods of α-Ag_2_WO_4_ can be observed across the entire surface of both samples.

**Figure 2:**
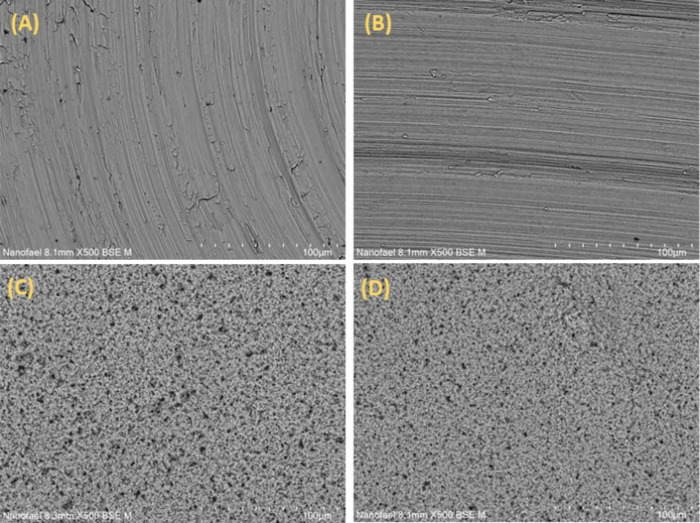
Scanning Electron Microscopy (SEM) images. Ti (A), Ti–I (B), Ti α-Ag_2_WO_4_ (C), Ti α-Ag_2_WO_4_–I (D).

EDS mapping was performed (Figure S2) that confirmed that the deposited particles were indeed composed of Ag, W, and O, and no significant differences were observed between the Ti α-Ag_2_WO_4_ and Ti α-Ag_2_WO_4_–I samples. Contact angle measurements were performed for all groups as significant changes were observed in the surface morphology of the Ti discs (Figure 3A–C). For Ti and Ti–I, all the wetting agents showed high contact angle values (Table S1). No statistical difference was observed between the Ti and Ti–I groups in the measurements with water and formamide, but a difference was observed with diiodomethane (*p* < 0.0001). When comparing the coated and uncoated discs, Ti α-Ag_2_WO_4_ and Ti α-Ag_2_WO_4_–I exhibited lower contact angles when liquids were applied to these surfaces (*p* < 0.0001), demonstrating increased wetting and confirming a shift toward hydrophilicity. The drop shape also visibly changed, supporting the contact angle measurements calculated using the Laplace–Young equation (Figure 3D).^55^

**Figure 3:**
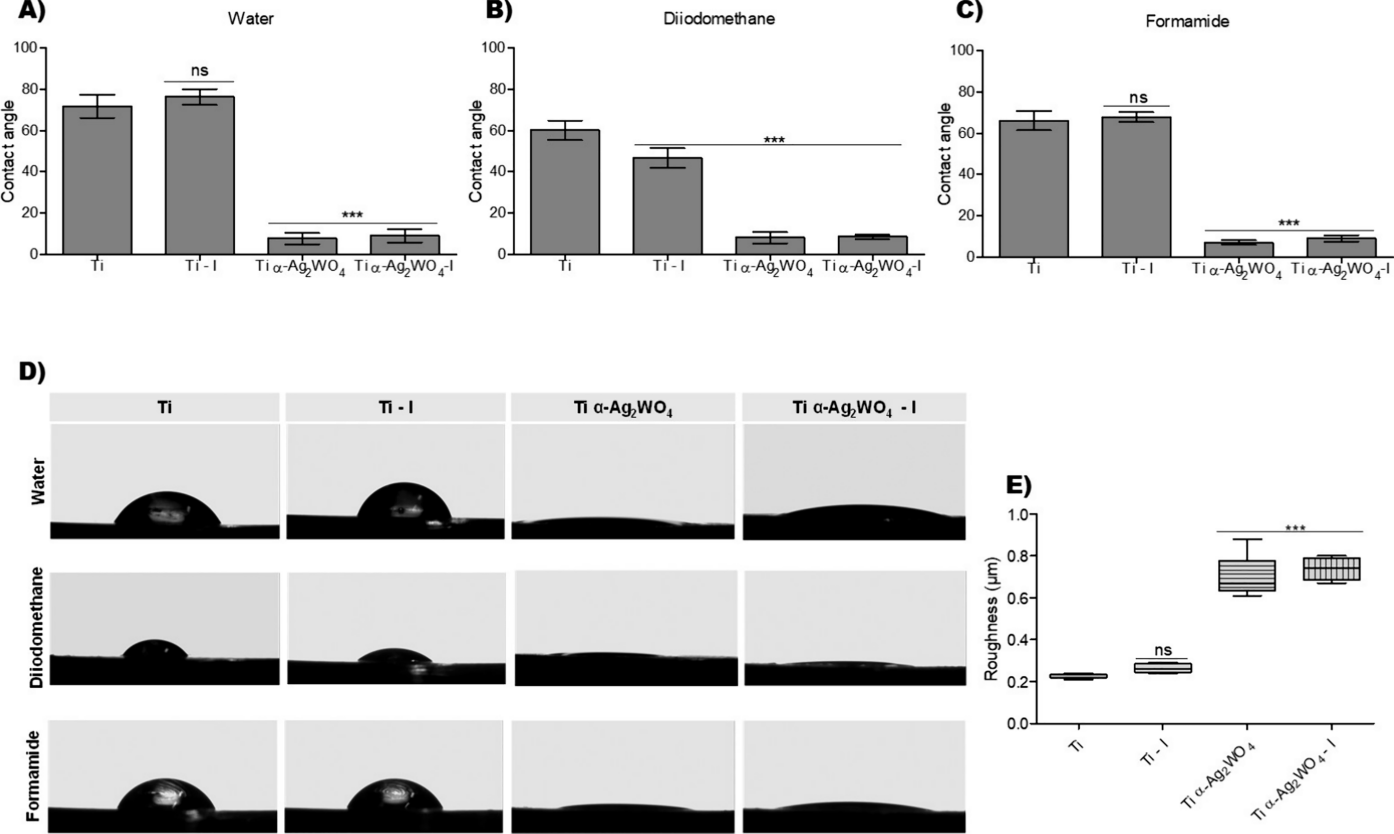
Surface characterization of Ti discs, either uncoated or coated with α-Ag_2_WO_4_. (A–C) Contact angles with water (A), diiodomethane (B), and formamide (C) (mean ± SD), analyzed by a one-way ANOVA followed by Tukey’s post hoc test. (D) Representative droplet images for the different groups (Ti, Ti–I, Ti-α-Ag_2_WO_4_, and Ti-α-Ag_2_WO_4_–I). (E) Boxplots of surface roughness (Ra, μm), analyzed by the Kruskal–Wallis test followed by Dunn’s post hoc test. ***p < 0.0001; ns: not significant; (*n* = 9).

Surface free energy was calculated according to the OWRK method using DataPhysics (Instruments GmbH, Filderstadt, Baden-Württemberg, Germany), which describes the polarity and dispersion contributions to the total surface energy. The surface free energy values obtained were 32.13 mN/m ± 4.11 for Ti, 35.02 mN/m ± 5.00 for Ti–I, 59.68 mN/m ± 0.46 for Ti α-Ag_2_WO_4_, and 61.38 mN/m ± 0.30 for Ti α-Ag_2_WO_4_–I. Similar values were observed between the Ti α-Ag_2_WO_4_ and Ti α-Ag_2_WO_4_–I discs (Figure S3). In regard to roughness, the Ti α-Ag_2_WO_4_ and Ti α-Ag_2_WO_4_–I discs presented a surface roughness (Ra) of 0.70 and 0.74 μm, respectively (Figure 3E). Thus, it can be observed that the deposited films increased the surface roughness that did not change with the irradiation of the discs. There was no statistical difference between Ti and Ti–I too.

The hydrophilic properties of the material surface are of great importance for adequate osseointegration of implants. In this work, the coatings rendered the Ti surface more hydrophilic, which is desirable in a clinical setting, as hydrophilic surfaces have improved interactions with biological fluids, cells, and tissues. Additionally, hydrophilic surfaces promote fibrin adhesion, which provides contact guidance for osteoblasts migrating along the surface, contributing to proper osseointegration.^66,67^ These findings are consistent with previous reports on Ag-containing coatings applied to Ti surfaces. Madiwal et al. (2024)^68^ modified Ti by Ag sputtering and observed a reduction in the contact angle and an increase in surface free energy, both proportional to the amount of Ag deposited, along with a concomitant improvement in cellular response and resistance to bacterial colonization. Surface roughness also influences implant osseointegration, contributing to firmer and faster osseointegration between the implant and the surrounding bone.^69,15^ In the present study, the addition of the coating increased the surface roughness of the Ti substrate, which will also be beneficial in a clinical setting. Similarly, in the study by Thukkaram et al. (2020),^70^ Ag-doped TiO_2_ coatings on Ti discs also exhibited increased surface roughness and wettability compared to untreated Ti substrates.

### 3.2 Antifungal and Antibacterial Activity

In recent years, several studies have focused on the development of Ti surface coatings to inhibit microbial adhesion and biofilm formation.^18,71–75^ Ag is an inorganic agent with antimicrobial properties, widely used in various fields, including medical implants.^76,77^ Ag can either be directly deposited onto Ti surfaces or added by growing a Ti oxide layer containing Ag to form coated surfaces.^72,29,31^ Furthermore, other strategies are being investigated, such as the incorporation of bioactive nanoparticles and polymers combined with photothermal therapy;^26^ the development of mesoporous silica nanoparticles loaded with antimicrobial peptides;^27^ biomimetic surfaces enriched with metal ions, such as copper;^28^ coatings containing redox-active nanoparticles capable of inducing ferroptosis;^30^ pH-sensitive coatings for controlled release of antimicrobial peptides;^32^ and MXene-class nanomaterials with antimicrobial and antiviral properties.^33^

Here, both unirradiated and irradiated coated discs (Ti α-Ag_2_WO_4_ and Ti α-Ag_2_WO_4_–I) exhibited significant antimicrobial activity, with reduced viability observed for all species. In the adhesion phase, reductions of 4.22, 1.69, 1.56, 4.27, 4.30, and 4.41 log_1__0_ CFU/mL were seen for *C. albicans*, *S. sanguinis*, *A. naeslundii*, *F. nucleatum*, and *P. gingivalis*, respectively, while in the biofilm phase, reductions of 6.69, 3.46, 3.31, 6.34, 6.40, and 7.38 log_1__0_ CFU/mL were seen for *C. albicans*, *S. sanguinis*, *A. naeslundii*, *F. nucleatum*, and *P. gingivalis*, respectively (*p* < 0.001 or 0.0001 for all species) (Figures 4 and 5A–E). For all species, Ti–I did not differ from the control (*p* > 0.05). These results were confirmed with CLSM, where coated discs showed predominantly red, PI-stained cells (dead cells) compared to the mostly green, Syto-9-stained cells (live cells) with the uncoated discs for all species (Figures 4 and 5F).

**Figure 4:**
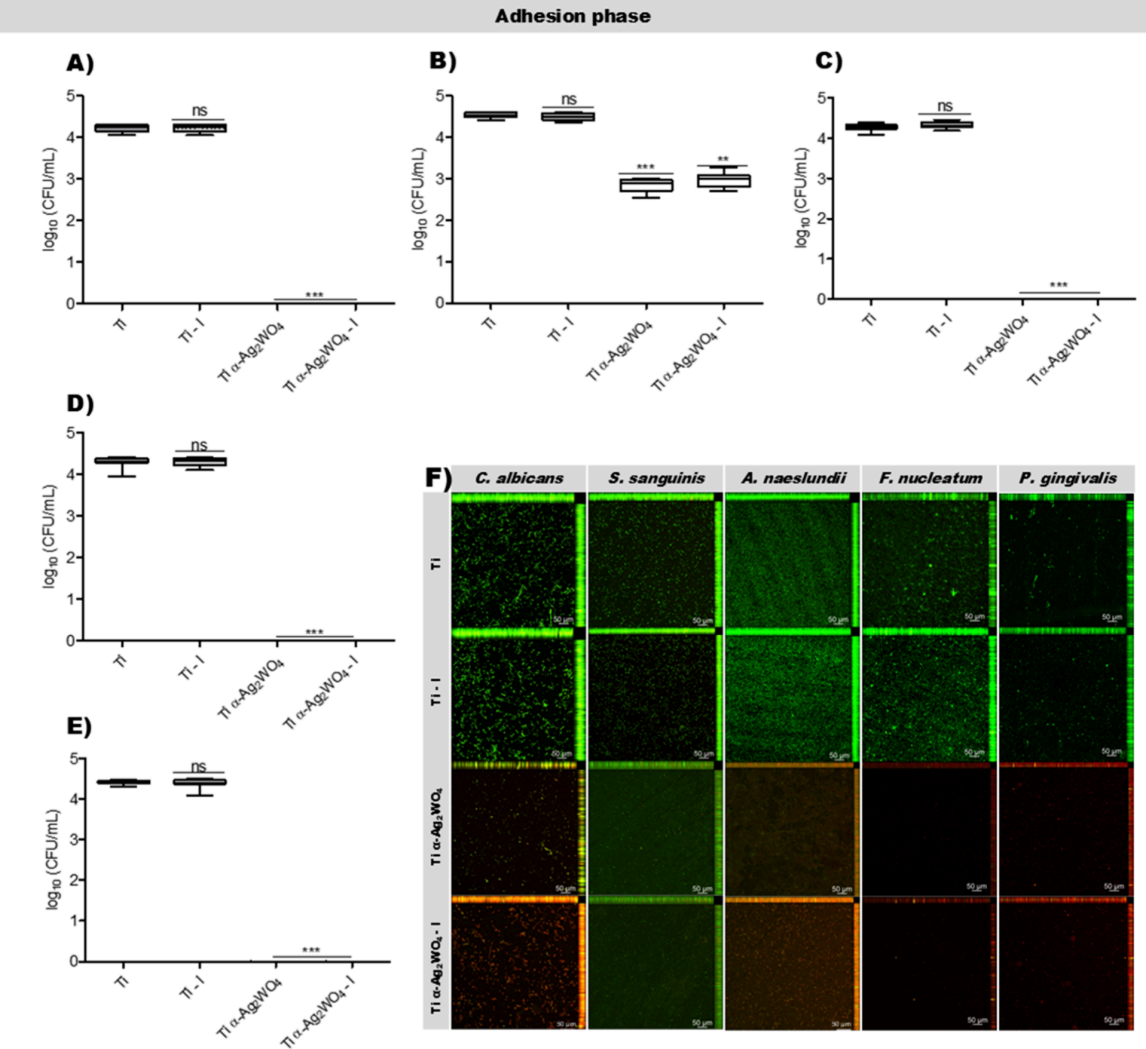
Antimicrobial effects of Ti α-Ag_2_WO_4_-coated or uncoated discs after adhesion. Boxplots showing log_1__0_ values of CFU/mL after the adhesion phase of *C. albicans* (A), *S. sanguinis* (B), *A. naeslundii* (C), *F. nucleatum* (D), and *P. gingivalis* (E). Statistical analysis was performed using the Kruskal–Wallis test followed by Dunn’s post hoc test. ** *p* < 0.001; *** *p* < 0.0001; ns: not significant; (*n* = 9). Representative CLSM images acquired with a 10× objective after microbial adhesion (F).

**Figure 5:**
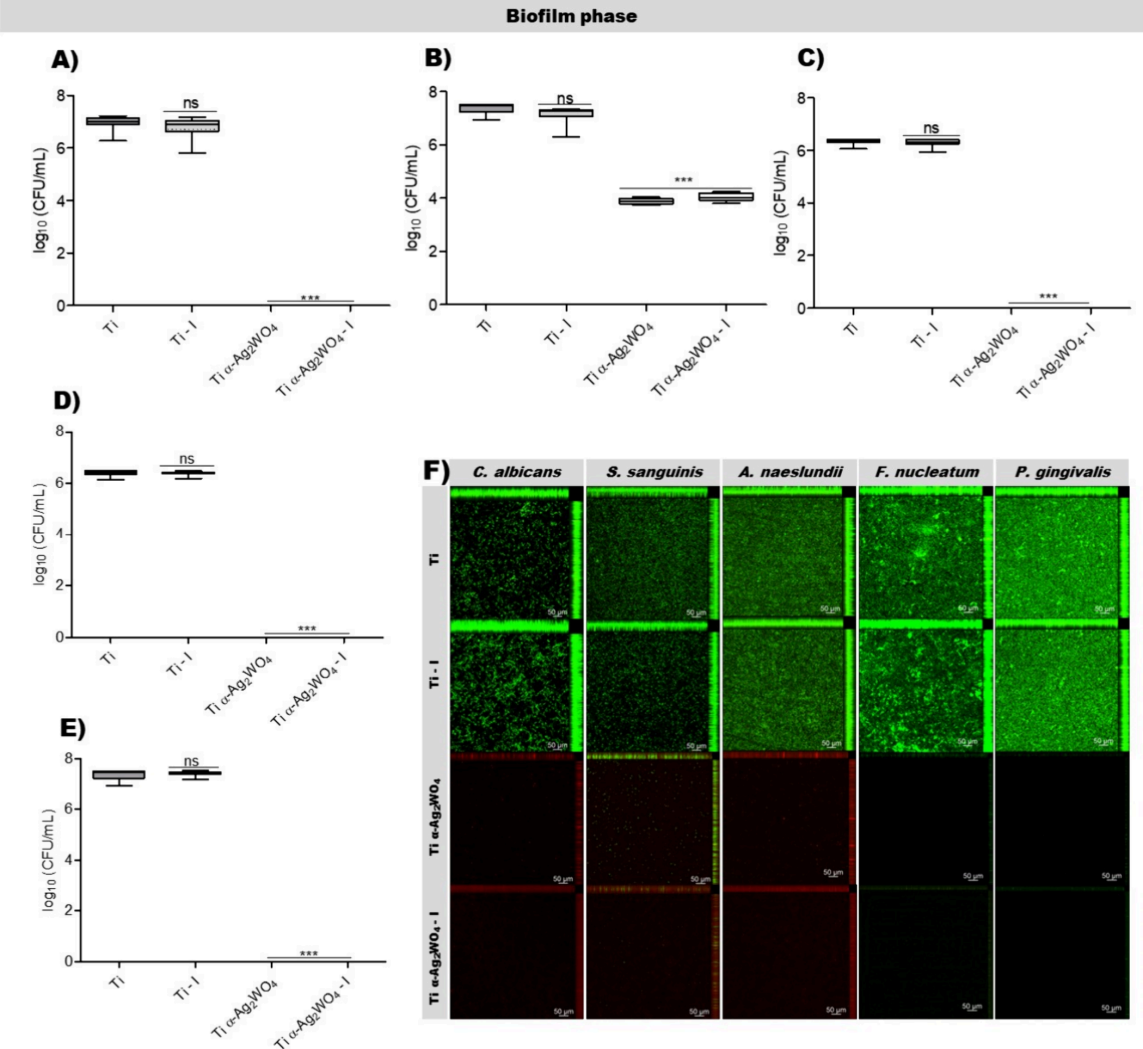
Antimicrobial effects of Ti discs α-Ag_2_WO_4_-coated or uncoated discs after biofilm formation. Boxplots showing log_1__0_ values of CFU/mL after the biofilm phase of *C. albicans* (A), *S. sanguinis* (B), *A. naeslundii* (C), *F. nucleatum* (D), and *P. gingivalis* (E). Statistical analysis was performed using the Kruskal–Wallis test followed by Dunn’s post hoc test. *** *p* < 0.0001; ns: not significant; (*n* = 9). Representative CLSM images acquired with a 10× objective after biofilm formation (F).

The antimicrobial effect observed in this study aligns with previous reports showing that incorporating Ag into Ti surfaces enhances antimicrobial activity. However, most previous studies focused only on the earlier stages of bacterial adhesion and biofilm formation. Juan et al. (2010),^78^ Xu et al. (2024),^79^ and Madiwal and Rajwade (2024),^68^ for instance, examined young biofilms and found antibacterial effects limited to short exposure periods. Choi et al. (2023)^29^ applied an aerosol-deposited coating and reported a reduction in bacterial biomass after 24–72 h, as measured by crystal violet staining, which is a technique that quantifies total biofilm mass but does not differentiate viable from nonviable cells. Similarly, van Hoogstraten et al. (2024)^80^ demonstrated biofilm inhibition in multilayer Ag coatings, though under conditions not representative of the oral environment. Regarding antifungal activity, D’Adamio et al. (2019)^81^ evaluated Ag-coated Ti against *Candida* spp. using the XTT assay, which estimates metabolic activity but cannot distinguish viable from metabolically active cells. In contrast, the present study investigated the coating’s effect on both initial bacterial adhesion and biofilm maturation, incorporating the prior formation of the acquired salivary pellicle as a natural substrate for microbial colonization. This experimental model, which more closely reproduces oral physiological conditions, provides stronger evidence of the coating’s antimicrobial efficacy and helps bridge existing gaps in the literature regarding the performance of Ag-containing coatings under realistic conditions.

The formation of biofilms on the surface of dental implant abutments is the main cause of peri-implant mucositis and peri-implantitis, where the soft tissue around the implant becomes inflamed.^82,83^ This inflammation promotes loss of alveolar bone and, therefore, the loss of the dental implants.^84,85^ There are several species associated with peri-implantitis. *P. gingivalis* is a pathogenic species directly related to the progression of peri-implantitis^86^ due to the numerous toxins produced^87^ as well as the strong inflammatory response induced.^88^ *F. nucleatum* is another microorganism strongly associated with peri-implantitis due to its ability to coaggregate with other species (including *P. gingivalis*) through the presence of multivalent adhesins in its cell wall.^16,89,90^ Furthermore, this bacterium can generate a capnophilic environment that allows the growth of anaerobic pathogenic bacteria.^91^ Additionally, the presence of *F. nucleatum* increases the invasion of *P. gingivalis* into host cells.^92^ Moreover, the formation of infectious processes in the oral cavity may be correlated with the development of systemic diseases, such as cardiovascular and pulmonary diseases with *S. sanguinis* and *P. gingivalis* infection,^93–95^ as well as acute appendicitis and colorectal cancer associated with *F. nucleatum*.^96,97^ Thus, the search for effective methods to control such bacterial species is of great relevance for the patient’s oral and systemic health.^98^

Biofilm formation is a multistage process that begins with the formation of a thin salivary layer on the surface of the implant.^99^ Afterward, the process continues with the adhesion of the initial colonizing microorganisms, including *S. sanguinis* and *A. naeslundii*, while the peri-implant pocket harbors both *P. gingivalis* and *F. nucleatum*. Once formed, the extracellular matrix provides protection for the microorganisms in internal layers, significantly increasing their tolerance to antimicrobial agents and, consequently, making microbial control difficult.^100^ Thus, once a mature biofilm has developed on any implant surface, eradicating the bacteria becomes highly challenging even with antibiotics, repeated surgical irrigation, and debridement. Poor vascularization, a high implant surface area, small colony variants, and sister cells through metabolic gene mutations are factors that make implant removal necessary whenever simple irrigation and debridement procedures fail to eradicate infections related to implanted devices. Additionally, whenever antibiotics are administered, the emergence of resistant strains poses an additional problem. To date, no treatment can guarantee the rapid and complete eradication of microbial biofilms or prevent reinfection. Therefore, long-term clinical success is associated with the antimicrobial properties of the implanted devices.^101^

Interestingly, in this study, *S. sanguinis* exhibited greater resistance to the α-Ag_2_WO_4_ coating than the other microorganisms tested, showing only partial reductions of 1.69 and 1.56 log_1__0_ (adhesion) and 3.46 and 3.31 log_1__0_ (biofilm) in the Ti α-Ag_2_WO_4_ and Ti α-Ag_2_WO_4_–I groups, respectively. In a previous study by our group,^102^ *S. aureus* also showed increased resistance to α-Ag_2_WO_4_ nanoparticles, with higher minimal inhibitory concentration (MIC) and minimal bactericidal/fungicidal concentration (MBC/MFC) values compared to *E. coli* and *C. albicans*. This difference in susceptibility was attributed to the distinct chemical composition of bacterial cell walls. *S. aureus*, a Gram-positive bacterium, possesses a thick peptidoglycan layer. Therefore, if nanoparticle-induced damage primarily occurs at the cell wall level, higher concentrations of nanoparticles may be required to inactivate this microorganism. However, in the present study, this difference in susceptibility cannot be attributed solely to Gram-positive classification, as *A. naeslundii*, which is also Gram-positive, showed a marked reduction in CFU/mL counts. The lower susceptibility of *S. sanguinis* is believed to be associated with the presence of a more efficient or better-regulated antioxidant system. Studies have shown that *S. sanguinis* expresses enzymes such as superoxide dismutase (SodA), peroxide response regulators (PerR), DNA protection proteins like Dps, and relies on manganese uptake via SsaACB to enhance its antioxidant defenses.^103,104^ Although *A. naeslundii* also expresses Mn/Zn superoxide dismutase and catalase,^105^ its antioxidant system appears to be less robust or less effective under the oxidative stress imposed by the microcrystal coating. Recent studies have explored the mechanisms by which *S. sanguinis* may exhibit increased tolerance to oxidative stress, which could partially explain the low reductions observed with the α-Ag_2_WO_4_ coating. For example, the SsaACB transporter is essential for maintaining intracellular manganese and iron pools and regulating redox homeostasis. Δ*ssaACB* mutant strains show reduced oxygen tolerance, impaired growth, and decreased activity of enzymes such as SodA and NrdEF, which depend on Mn as a cofactor.^104,106^ Furthermore, ZIP-type proteins have also been identified as an alternative pathway for Mn uptake, particularly in strains where SsaACB is absent or poorly active, indicating functional redundancy to ensure Mn availability under adverse conditions such as acidic pH or oxidative stress.^107^ In another study, *spxA1* mutants showed reduced tolerance to H_2_O_2_ and other oxidative stresses, indicating that this regulatory pathway is crucial for the microorganism’s resistance.^108^ These findings suggest that *S. sanguinis* possesses a more efficient or tightly regulated antioxidant system, possibly associated with Mn transport and the maintenance of redox enzymes, which confers an advantage against damage induced by microcrystals or nanoparticles. Future studies could directly assess the expression of SodA, NrdEF, and ZIP transporters, as well as evaluate mutants or inhibitors of these transporters, to better elucidate the mechanisms underlying these differences in susceptibility.

### 3.3 Virucidal Activity and Vero E6 Viability

To investigate the virucidal properties of **Ti**, **Ti–I, Ti α-Ag**_**2**_ **WO**_**4**_, and **Ti α-Ag**_**2**_ **WO**_**4**_ **-I**, we conducted a series of experiments measuring their effects on SARS-CoV-2 particles over varying interaction durations (5, 20, 40, and 60 min). Viral titers were assessed after virucidal assay, and the results were as follows: Ti and Ti–I discs demonstrated titers comparable to the control (Figure 6A). Conversely, Ti α-Ag_2_WO_4_ and Ti α-Ag_2_WO_4_–I discs exhibited significant virucidal activity, initiating damage to the SARS-CoV-2 particles after 20 min of exposure. This interaction led to a decrement of 1 log_10_ in viral titers. Notably, Ti α-Ag_2_WO_4_ disc at 60 min postinteraction displayed the highest efficacy, achieving a reduction of 2 log_10_ in viral titers. The comparison between irradiated and nonirradiated discs, as well as between Ti discs coated or not with α-Ag_2_WO_4_, revealed no significant differences, except in cases involving Ti or Ti -I at the 40 and 60 min e intervals, where they exhibited an incremental reduction of 1-log_10_ and 2-log_10_, respectively, compared to the Ti α-Ag_2_WO_4_ disc (Figure 6A). It is important to note that the observed antiviral effects were attributed solely to the direct interaction of the discs with the virus, as confirmed by the lack of detrimental impact on Vero E6 cell viability (Figure 6B).

**Figure 6:**
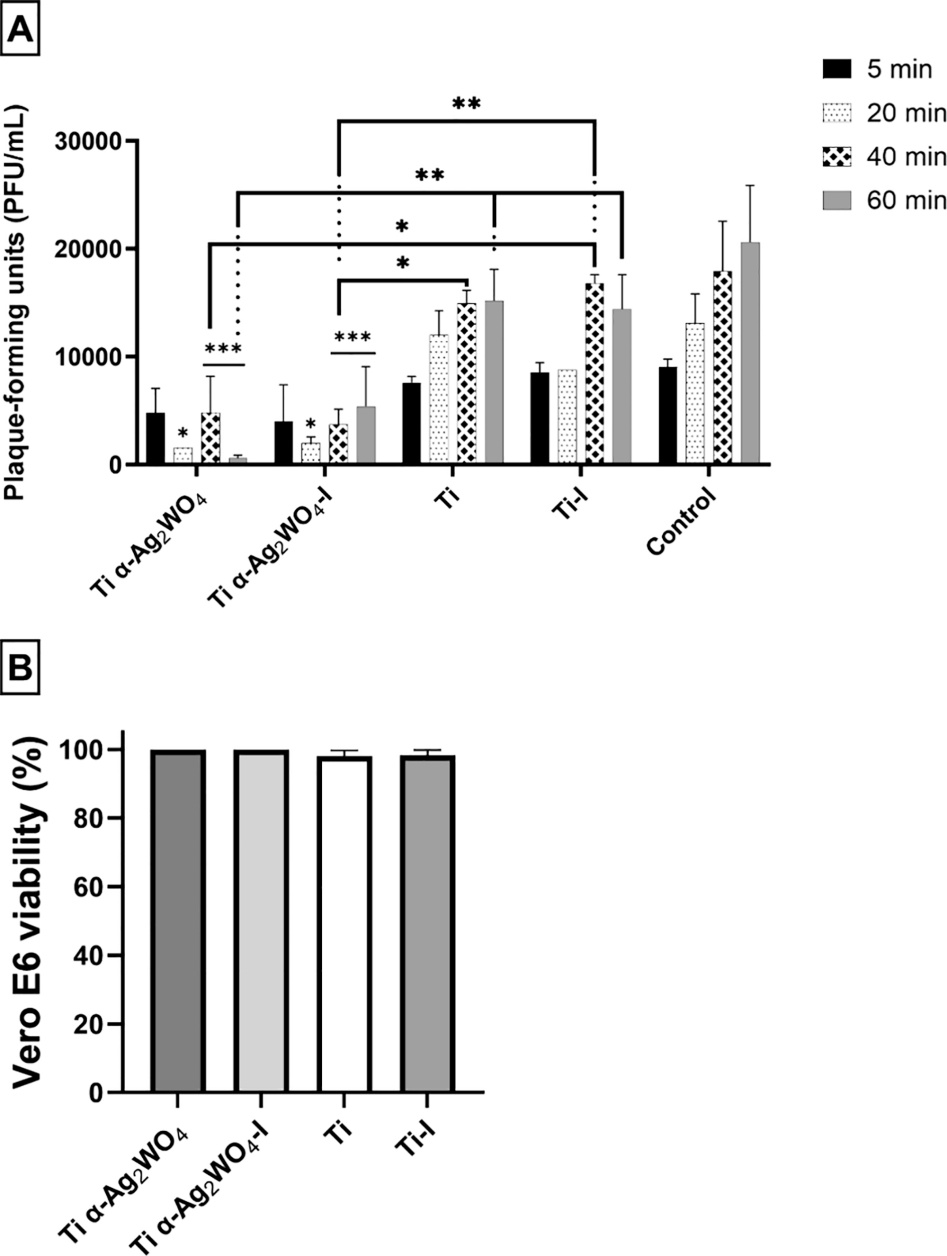
Virucidal effects of Ti discs coated or not with α-Ag_2_WO_4_ on SARS-CoV-2 infection and impact on Vero E6 cell viability. (A) The virus was incubated in the medium alone (control) or with Ti discs (Ti, Ti–I, Ti-α-Ag_2_WO_4_, Ti-α-Ag_2_WO_4_–I) for 5, 20, 40, and 60 min. Viral infectivity was assessed in Vero E6 cells by plaque assay (PFU/mL, *n* = 3). Statistical analysis was performed using two-way ANOVA followed by Tukey’s post hoc test (*p ≤ 0.05, **p < 0.01, ***p < 0.001). (B) Supernatants collected after 60 min were used to treat Vero E6 cells for 72 h, and cell viability was measured using the MTT assay (*n* = 3). Statistical analysis was performed using one-way ANOVA followed by Tukey’s post hoc test versus the MOCK group (100% viability).

#### 3.3.1 SARS-CoV-2 Morphology

Examination of the SARS-CoV-2 control sample, consisting of untreated virus, revealed particles maintaining their characteristic morphology (Figure 7A). SARS-CoV-2 particles after treatment with Ti discs (Figure 7B) and Ti–I discs (Figure 7C–D) showed unchanged viral particles, while defective particles were observed in the samples treated with Ti α-Ag_2_WO_4_ (Figure 7E) and Ti α-Ag_2_WO_4_–I discs (Figure 7F).

**Figure 7:**
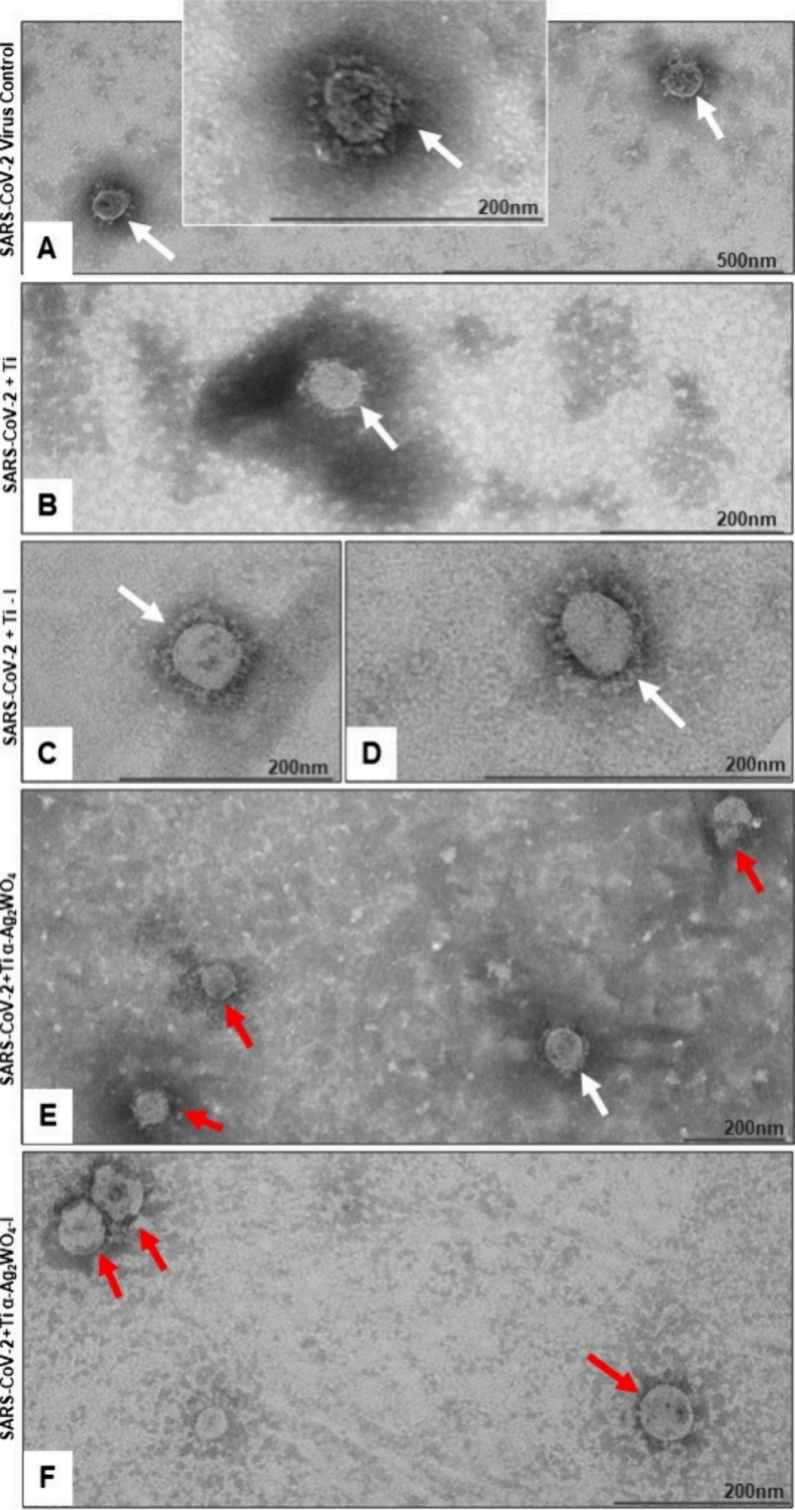
Ultrastructural analysis of SARS-CoV-2 particles in the presence of irradiated and nonirradiated Ti and Ti coated with α-Ag_2_WO_4_ discs. (A) Control particles showing spherical morphology with characteristic spikes (white arrows). (B–D) Particles treated with Ti or irradiated Ti maintain intact morphology (white arrows). (E, F) Particles treated with Ti α-Ag_2_WO_4_ or irradiated Ti α-Ag_2_WO_4_-I exhibit defective morphology (red arrows).

The findings from the ultrastructural analysis are consistent with the data obtained from both the viral suspension titrations and the virucidal assays. The viral titers in the suspensions showed a pattern similar to that observed in the virucidal assay results, in which the Ti α-Ag_2_WO_4_ discs demonstrated the most significant reduction in log values. Contrary to the results from the virucidal assays employing a viral input of 10^4^ PFU/mL, a statistical difference was observed between the Ti disc and Ti–I disc when compared to the control (Figure 8). This discrepancy is likely attributable to the elevated viral titers utilized in this assay, necessitating the use of 10^6^ PFU/mL to facilitate the detection of viral particles via negative staining techniques.

**Figure 8:**
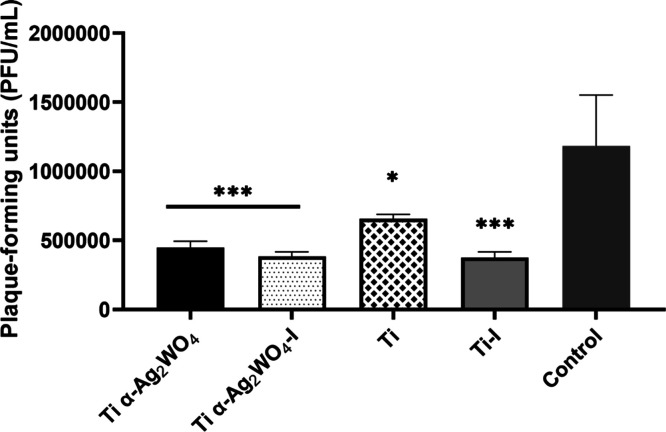
Assessment of viral titers following exposure to metal discs in TEM assays. SARS-CoV-2 (10^6^ PFU/mL) was incubated for 60 min in the medium alone (control) or with uncoated Ti, irradiated Ti (Ti–I), Ti coated with α-Ag_2_WO_4_ (Ti α-Ag_2_WO_4_), and coated plus irradiated Ti (Ti α-Ag_2_WO_4_–I) discs. Viral titers were assessed by plaque assay (PFU, *n* = 3). Statistical analysis was performed using one-way ANOVA followed by Tukey’s post hoc test (*p ≤ 0.05, ***p < 0.001).

The effect of α-Ag_2_WO_4_ microcrystals against SARS-CoV-2 was also reported in the study by Pereira et al. (2022),^46^ in which the authors evaluated chitosan/α-Ag_2_WO_4_ (CS6.6/α-Ag_2_WO_4_) composites synthesized by femtosecond laser irradiation. In particular, the CS6.6/α-Ag_2_WO_4_ composite reduced the SARS-CoV-2 viral titer by up to 80% compared with control samples, an effect attributed to the generation of ROS that inactivate the virus upon contact. Similarly, Assis et al. (2022)^109^ synthesized α-Ag_2_WO_4_ and immobilized it on polypropylene, achieving an antiviral efficiency greater than 98% after only 10 min of contact with the material. Our results demonstrated that the coating, with or without irradiation, was not cytotoxic to Vero E6 cells. Previous studies have also confirmed the biocompatibility of α-Ag_2_WO_4_, showing that this microcrystal does not affect the proliferation or mitochondrial enzymatic activity of fibroblasts cultured either in monolayers or within a collagen matrix.^49,50^ These findings emphasize the potential of α-Ag_2_WO_4_-functionalized Ti surfaces as effective, nontoxic antiviral platforms for dental and medical devices, particularly in applications requiring the rapid inactivation of enveloped viruses such as SARS-CoV-2.

This study highlights spin coating as a promising method for functionalizing Ti surfaces with α-Ag_2_WO_4_, emphasizing its advantages such as simplicity, low cost, reproducibility, uniformity, and scalability for biomedical implants.^35^ Previous studies have demonstrated that this approach enables the production of uniform, bioactive, antimicrobial, and biocompatible coatings under different conditions and on various substrates.^36,37,110^ More recently, strategies such as pH-responsive coatings prepared by spin coating have further reinforced its potential.^32^ The present work uniquely integrates the evaluation of both antimicrobial activity against clinically relevant oral microorganisms and the antiviral activity of Ti coatings with α-Ag_2_WO_4_ obtained by spin coating, underscoring its translational relevance.

## 4 Conclusions

In conclusion, the coating of Ti with α-Ag_2_WO_4_ is a promising alternative for the development of dental implant abutments and other Ti devices used in the biomedical field. The coating promoted an increase in the roughness and hydrophilicity of the material, which will contribute to adequate osseointegration of the device. In addition, the coating proved to be biocompatible and effective in controlling the formation of biofilms of *A. naeslundii*, *S. sanguinis*, *P. gingivalis*, *F. nucleatum*, and *C. albicans*, as well as exhibiting anti-SARS-CoV-2 properties. However, EBI irradiation did not provide additional benefits in the antimicrobial activity of the material. Future studies should be conducted to better mimic the complex oral microenvironment using polymicrobial biofilm models. Furthermore, it will be important to evaluate the long-term biocompatibility of the coatings, as well as their stability, controlled ionic release, and potential cumulative cytotoxic effects on human cells. Finally, investigating the need for reapplication or maintenance of the coatings’ functionality over time, especially considering wear and tear from clinical use, is also highly relevant.

## Supplementary Material



## Notes

Raman modes of nonirradiated and irradiated Ti discs (Figure S1); chemical composition mapping of the samples by SEM equipped with energy-dispersive spectroscopy (EDS) (Figure S2); mean contact angle measurements (Table S1); surface free energy of Ti discs, coated or uncoated with α-Ag_2_WO_4_, and an image illustrating the surface free energy of wetting agents on the discs (Figure S3) (PDF)

## Abbreviations

ANOVA: analysis of variance
Ag: silver
ATCC: American Type Culture Collection
BHI: brain heart infusion agar
CCD: charge-coupled device
CFU/mL: colony-forming units per milliliter
CLSM: confocal laser scanning fluorescence microscopy
DMEM: Dulbecco’s modified Eagle’s medium
EBI: electron beam irradiation
EDS: energy-dispersive X-ray spectroscopy
FAB: fastidious anaerobe broth
FBS: fetal bovine serum
MBC: minimum bactericidal concentration
MFC: minimum fungicidal concentration
MIC: minimum inhibitory concentration
MTT: 3-(4,5-dimethylthiazol-2-yl)-2,5-diphenyltetrazolium bromide
MSSA: methicillin-susceptible *Staphylococcus aureus*
OWRK: Owens–Wendt–Rabel–Kaelble
PBS: phosphate-buffered saline
PFU/mL: plaque-forming units per milliliter
PI: propidium iodide
PTA: phosphotungstic acid
ROS: reactive oxygen species
RPMI: Roswell Park Memorial Institute
SDA: sabouraud dextrose agar
SDS: sodium dodecyl sulfate
SEM: scanning electron microscopy
TEM: transmission electron microscopy
Ti: titanium
TSB: tryptic soy broth
WHO: World Health Organization
XRD: X-ray diffraction
YNB: yeast nitrogen base broth.
