# Supplementary material for: Antimicrobial and Antiviral Activities, and Biocompatibility of Titanium Coated with Electron Beam-Irradiated α‑Silver Tungstate Microcrystals
Source: ACS Omega. 2025 Nov 23;10(48):58975–90. doi: 10.1021/acsomega.5c07850 (PMC12771465; doi:10.1021/acsomega.5c07850)
Supplement: Supplementary file 1 [file ao5c07850_si_001.pdf]

Supporting Information

Antimicrobial and Antiviral Activities, and Biocompatibility of Titanium Coated with Electron Beam–Irradiated α-Silver Tungstate Microcrystals

Sarah Raquel de Annunzio[A], Marcelo Assis[B], Paula Aboud Barbugli[A], Alice Santos Rosa[C], Thamara Kelcya Fonseca Oliveira[C], Tayane Alvites Nunes Quintão[C], Vivian Neuza dos Santos Ferreira[C], Thayane da Encarnação Sá-Guimarães [C], Giovanna Barbosa da Conceição[C], Débora Ferreira Barreto-Vieira[C], Rodolfo Debone Piazza[D], Rodrigo Fernando Costa Marques[D], Milene Dias Miranda[C], Elson Longo[E], Carlos Eduardo Vergani[A]*

[A]Departamento de Materiais Odontológicos e Prótese, Faculdade de Odontologia, Universidade Estadual Paulista "Júlio de Mesquita Filho" (UNESP), Araraquara, SP, 14801-903, Brazil.

[B]Departamento de Biociências, Universidade Federal de São Paulo (UNIFESP), Santos, SP, 11015-020, Brazil.

[C]Laboratório de Morfologia e Morfogênese Viral, Instituto Oswaldo Cruz, Fundação Oswaldo Cruz, RJ, 21040-900, Brazil.

[D]Departamento de Química Analítica, Físico-Química e Inorgânica, Instituto de Química, Universidade Estadual Paulista "Júlio de Mesquita Filho" (UNESP), Araraquara, SP, 14800-060, Brazil.

[E]CDMF, Universidade Federal de São Carlos (UFSCar), São Carlos, SP, 13565-905, Brazil.

***Corresponding author:** carlos.vergani@unesp.br, phone: +55 16 3301-6542

KEYWORDS: silver tungstate microcrystals, coating, titanium, antimicrobial activity, antiviral activity, peri-implantitis

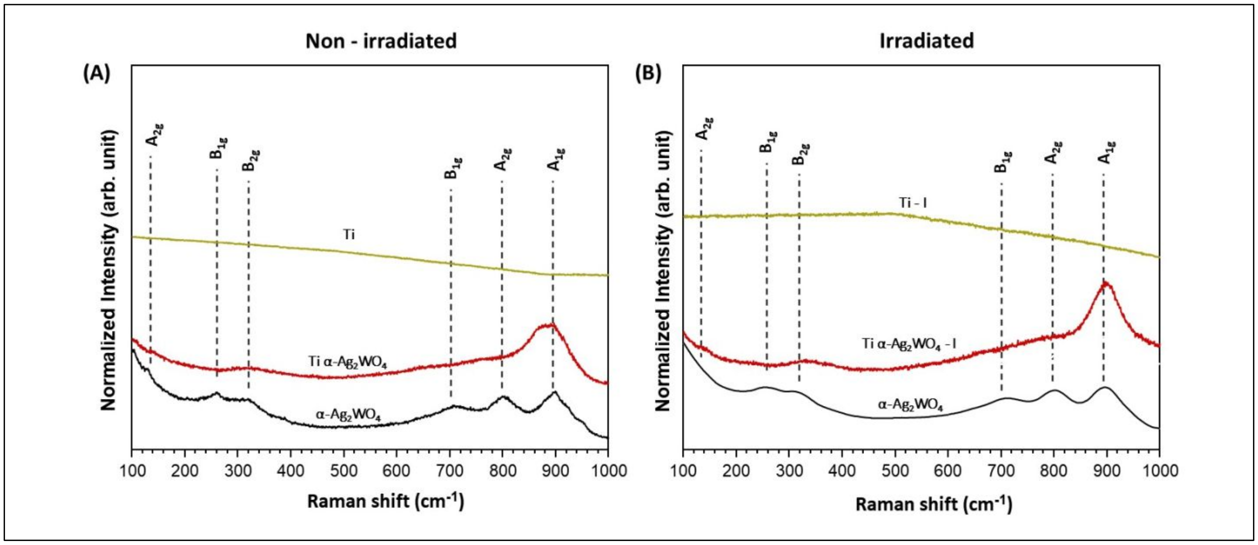

Figure S1. Raman modes for non-irradiated **(A)** and irradiated Ti discs **(B)**.

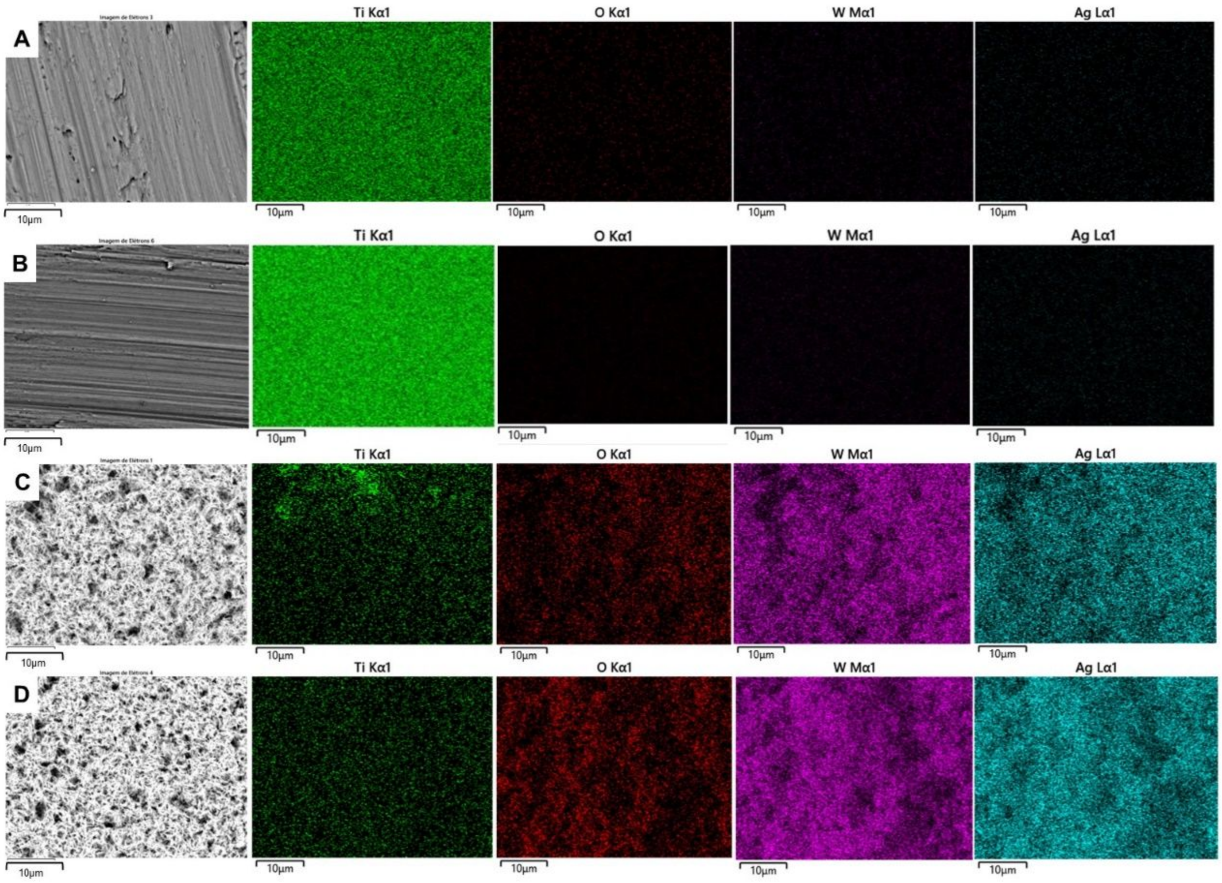

Figure S2. Mapping of the chemical composition of the samples by SEM equipped with an energy dispersive spectroscopy (EDS) detector. Line A: Ti, B: Ti-I, C: Ti α-Ag_2WO_4, D: Ti α-Ag_2WO_4-I. Markings in green: Ti, red: O, pink: W and blue: Ag.

Table S1. Contact angle measurements. Mean and standard deviation values of contact angle measurements for the three wetting agents (n = 9). Ti (control), Ti-I, Ti α-Ag_2WO_4 and Ti α-Ag_2WO_4-I.

**Wetting agents**	**Samples**			
	**Ti**	**Ti-I**	**Ti α-Ag_2WO_4**	**Ti α-Ag_2WO_4-I**
Water	71.79 ± 5.55	76.37 ± 3.82	7.72 ± 2.77	9.11 ± 3.23

Diiodomethane	60.22 ± 4.77	46.79 ± 4.76	8.10 ± 2.67	8.52 ± 0.98
Formamide	66.21 ± 4.67	67.97 ± 2.45	7.10 ± 0.99	8.55 ± 1.07

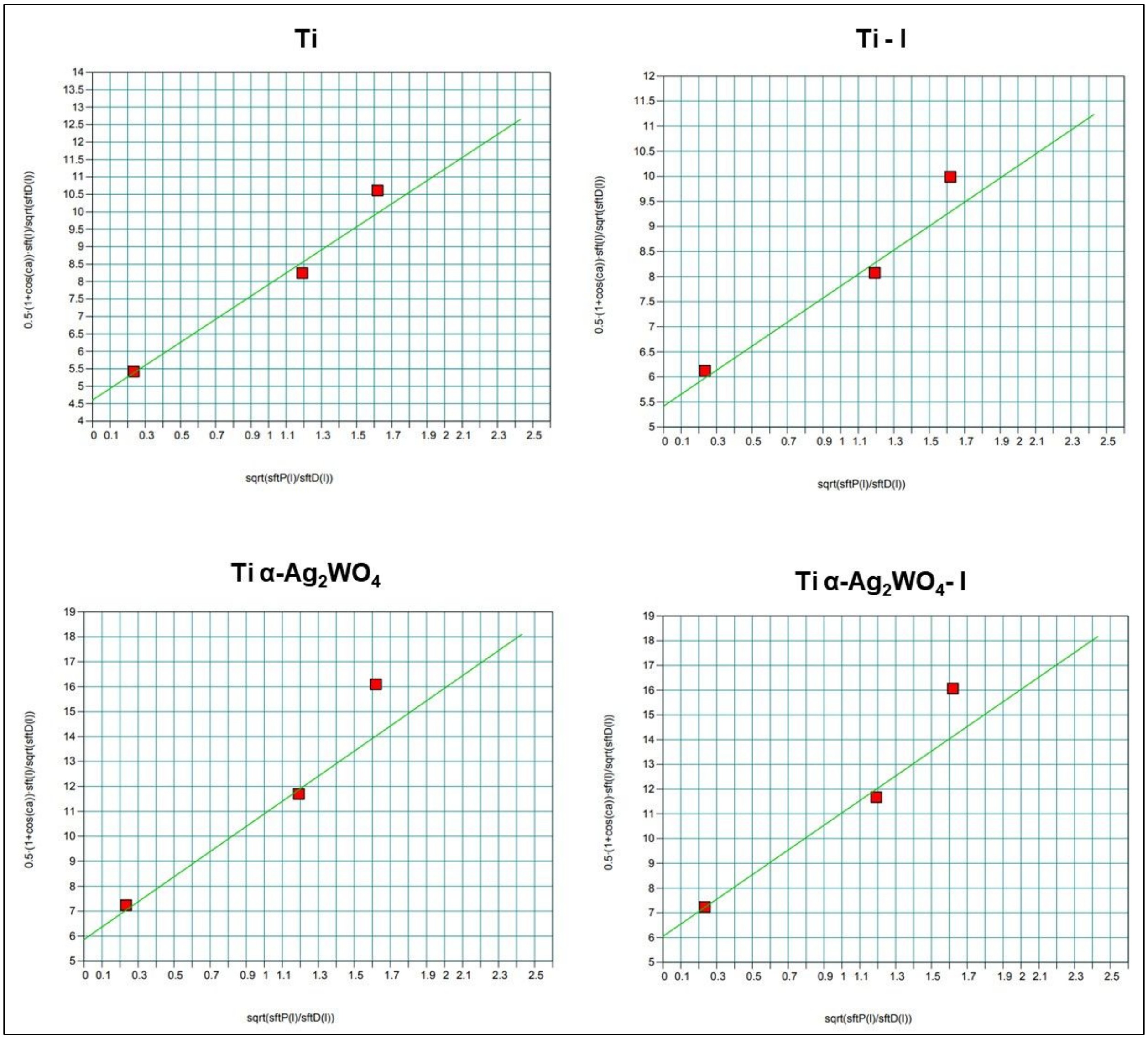

Figure S3. Surface free energy of Ti discs coated or not with α-Ag_2WO_4. Image demonstrating the surface free energy of the wetting agents on the discs of Ti, Ti-I, Ti α-Ag_2WO_4 and Ti α-Ag_2WO_4-I.
